# E‐cigarette or vaping product use‐associated lung injury in an adolescent

**DOI:** 10.5694/mja2.51462

**Published:** 2022-03-24

**Authors:** Cameron RL McKenzie, Joshua Davis, Adrian J Dunlop

**Affiliations:** ^1^ University of Newcastle Newcastle NSW; ^2^ John Hunter Hospital Newcastle NSW; ^3^ Hunter New England Local Health District Newcastle NSW

**Keywords:** Respiratory distress syndrome, Lung diseases, Electronic cigarettes, Smoking, Toxicology

## Competing interests

No relevant disclosures.

to the editor: Chan and colleagues^1^ recently reported a case of putative e‐cigarette or vaping product use‐associated lung injury (EVALI) in a 15 year‐old girl who was a low level user of vaporised nicotine (without adulterants). We believe that, rather than EVALI, her presentation is better explained by urosepsis‐related acute lung injury.

Current guidance from the United States Centers for Disease Control and Prevention (CDC)^2^ emphasises the role of adulterants, especially vitamin E acetate, in EVALI. In a US study completed before the widespread adoption of e‐cigarettes, the incidence of acute lung injury in 15–19‐year‐olds was 16 per 100 000 patient‐years, with many cases stemming from non‐pulmonary sepsis.^3^ In February 2020, only 2807 cases of vaping lung injury had been reported in the US, representing an incidence of well under one case per 100 000 patient‐years.^1^

Given these rates, as well as the patient’s prominent dysuria, polyuria, back pain and worsening pyrexia, we think urosepsis triggered the acute lung injury in this case. The authors say that sepsis was ruled out due to negative blood and urine cultures. However, if samples were collected after the initiation of antibiotics, false negative cultures are common in sepsis.

The patient met the accepted criteria for sepsis, with suspected infection, a systemic inflammatory response syndrome and acute end‐organ failure,^4^ and was treated for this condition with antibiotics and corticosteroids for the acute lung injury.

The CDC criteria for EVALI emphasise that the diagnosis should only be made where there is “no evidence in [the] medical record of alternative plausible diagnoses”.^5^ Dysuria, polyuria and back pain are not known symptoms of EVALI, and the authors have not explained how EVALI could account for this aspect of her presentation nor why these symptoms preceded the respiratory symptoms. In conclusion, the evidence to support a diagnosis of EVALI is insufficient in this case, and an alternative explanation is far more likely. Therefore, this case report should not be regarded as evidence for a case of EVALI occurring in Australia.

## References

[1] Chan BS , Kiss A , McIntosh N , et al. E‐cigarette or vaping product use‐associated lung injury in an adolescent. Med J Aust 2021; 215: 313‐314. https://www.mja.com.au/journal/2021/215/7/e‐cigarette‐or‐vaping‐product‐use‐associated‐lung‐injury‐adolescent 34490629 10.5694/mja2.51244

[2] Centers for Disease Control and Prevention . Outbreak of lung injury associated with the use of e‐ cigarette, or vaping, products. https://www.cdc.gov/tobacco/basic_information/e‐cigarettes/severe‐lung‐disease.html (viewed Oct 2021).

[3] Rubenfeld GD , Caldwell E , Peabody E , et al. Incidence and outcomes of acute lung injury. N Engl J Med 2005; 353: 1685‐1693. 16236739 10.1056/NEJMoa050333

[4] Singer M , Deutschman CS , Seymour CW , et al. The third international consensus definitions for sepsis and septic shock (Sepsis‐3). JAMA 2016; 315: 801‐810. 26903338 10.1001/jama.2016.0287 PMC4968574

[5] Centers for Disease Control and Prevention . 2019 Lung injury surveillance primary case definitions — September 18, 2019. https://www.cdc.gov/tobacco/basic_information/e‐cigarettes/assets/2019‐Lung‐Injury‐Surveillance‐Case‐Definition‐508.pdf (viewed Oct 2021).

